# A Novel Water Method for Reducing Air Conduction in Soft Tissue Conduction

**DOI:** 10.3390/audiolres16020041

**Published:** 2026-03-07

**Authors:** Shai Chordekar, Haim Sohmer, Miriam Geal-Dor

**Affiliations:** 1 Department of Communication Disorders, Ariel University, Ariel 4070000, Israel; shaic@ariel.ac.il; 2 Department of Medical Neurobiology (Physiology), Hebrew University-Hadassah Medical School, Jerusalem 9112102, Israel; haims@ekmd.huji.ac.il; 3 Speech & Hearing Center, Hebrew University-Hadassah Medical Center, Jerusalem 9112001, Israel; 4 Department of Communication Disorders, Jerusalem Multidisciplinary College, Jerusalem 9422408, Israel

**Keywords:** soft tissue, water, air conduction, acoustic impedance, vibration, bone vibrator

## Abstract

**Background:** Bone vibrator (BV) stimulation applied to skin sites on the body elicits hearing by soft tissue conduction (STC). However, BV stimulation to sites far from the ear requires the delivery of higher-intensity stimulus vibrations to achieve threshold, which can then induce hearing by air conduction (AC) contamination. This problem limits the study of STC thresholds at sites more distant from the ear. **Objective:** To overcome this problem, we evaluated the possibility of delivering STC vibratory stimuli to body sites in a water bath, based on the different acoustic impedances between air and water, which produces a 30 dB reduction in transmission from water to air. **Methods:** A standard clinical BV delivered vibration stimuli (tonal and speech stimuli) applied directly to two body sites: finger and foot. BV and body sites were immersed in a water bath. One control involved both stimulation site and BV both in water, but not in contact. In an additional control, the BV was in the bath, while the stimulation site was out of the bath. **Results:** STC hearing of both pure tones and speech could be elicited at stimulus intensities below those induced by control stimulation (body site and BV both in water, but not in contact; BV in bath, stimulation site out of bath). STC thresholds at the finger site were lower than those at the foot. **Conclusions:** The current results suggest that water-immersion method enables study of STC hearing in response to higher-intensity vibrational stimuli, and at body sites more distant from the ear, without contamination by AC hearing.

## 1. Introduction

Soft-tissue conduction (STC) refers to the auditory sensation elicited when vibratory stimuli are applied to skin over soft tissue sites, representing a third pathway for sound perception alongside the more familiar pathways of air conduction (AC) and bone conduction (BC) [1]. It is proposed that STC generates vibrations within the body’s tissues, such as skin and muscle, which then travel through different soft tissue structures, ultimately reaching and activating the inner ear. This pathway is typically tested by placing a vibrating device, usually the clinical bone vibrator (BV), on body areas that do not directly overly the skull [2,3,4,5,6,7,8,9,10,11]. Research has confirmed that these vibrations can travel through body tissues to activate the inner ear and continue along the auditory pathway to the brain, creating a sensation of hearing [12].

One key area of interest in this field is “distal” stimulation, applying vibrations to body regions distant from the head, such as the foot or ankle [3,6,13,14]. Since these stimulation sites are far from the head and inner ear, the resulting sound waves must travel through multiple body tissues over a long distance before reaching the cochlea.

However, this presents a significant challenge: as vibrations travel toward the ear, they are naturally reduced in intensity according to the inverse-square law [15]. Consequently, eliciting a response from the foot requires very high-intensity stimulation [3,6]. At these levels, the vibrating device inadvertently functions as a loudspeaker, producing airborne sound that reaches the ear via air conduction. This documented “air-conduction contamination” complicates the isolation of pure soft-tissue hearing thresholds and underscores the need for an effective method to mitigate airborne interference [3,6,14].

The inability to distinguish pure soft-tissue hearing from air-conduction contamination has limited the clinical utility of distal stimulation; addressing this gap is critical for developing accessible diagnostic tools that can accurately map the body’s auditory conductivity.

To address this challenge, we investigated a method that utilizes water as an acoustic barrier to minimize unwanted AC sound. This approach exploits the physical principle of acoustic impedance, defined as the product of the density of the medium and the velocity of sound in that medium [16]. The significant impedance mismatch at the water–air interface, arising from the vast disparity in acoustic impedance between these two media, causes the majority of acoustic energy to be reflected rather than transmitted. Theoretically, approximately 99.9% of the vibration magnitude is reflected at this boundary, resulting in an estimated transmission loss of 30 dB [16,17,18]. By immersing both the vibrating device and the distal body site in a water bath, this setup serves as an acoustic shield, allowing vibrations to reach the surface of the body (skin, soft tissue) and to propagate through the tissues of the body, while significantly attenuating the airborne sound that would otherwise reach the ears. The practical utility of this approach lies in its use of standard clinical equipment, such as the Radioear B71 BV, which offers a feasible alternative to the specialized and expensive high-intensity transducers typically required for distal stimulation. These results suggest that STC can be evaluated with reduced airborne interference in a standard clinical environment, thereby aiding our understanding of sound propagation through the body. Beyond basic research, this technique may eventually assist in refining diagnostic protocols for conditions such as Superior Canal Dehiscence Syndrome (SCDS), where patients often exhibit hypersensitivity to vibrations from distal body sites [13,19]. The primary objective of this study, therefore, was to evaluate a water-immersion method designed to isolate distal STC thresholds, by reducing the effects of AC contamination. This would allow the study of STC thresholds at more distant sites on the body.

## 2. Materials and Methods

### 2.1. Participants

Ten healthy subjects (2 males, 8 females; mean age ± standard deviation: 24.6 ± 2.7; range 21 to 28 years, mean height ± standard deviation: 165.2 ± 11.1; range 160 to 188 cm) participated in the study. All participants demonstrated normal hearing, defined as AC thresholds at or better than 15 dB HL at the frequencies of 0.5, 1.0, 2.0, and 4.0 kHz, with no history of ear infections, operations, or other otological pathologies. The study protocol was reviewed and approved by the Jerusalem Multidisciplinary College Ethics Committee (no. 667-2025), and all subjects provided written informed consent prior to participation.

### 2.2. Instrumentation and Setup

Testing was conducted in a standard sound-proof audiometric booth using a Grason Stadler Inc. AudioStar Pro clinical audiometer (AudioStar Pro^TM^, GSI., Eden Prairie, MN, USA) calibrated according to American National Standards Institute standard [20]. All sessions were carried out by the same two testers to ensure consistency [21]. Vibratory stimuli were delivered via a Radioear B71 Bone conducter (Radioear, Pittsburgh, PA, USA). To prevent electrical short-circuiting during water immersion, the BV was first wrapped in cling film and then sealed within a plastic Ziplock bag. The immersion setup consisted of a round water bath with a diameter of 32 cm and a depth of 14 cm, filled with water to a level of 10 cm. Throughout the experimental procedures, participants were equipped with deeply inserted earplugs (Classic SuperFit 30 AeroCo, E-A-R, Indianapolis, IN, USA) and high-attenuation earmuffs (3M Peltor X5A earmuffs hearing protector, St. Paul, MN, USA) to isolate the STC pathway and minimize the detection of any residual airborne sound produced by the BV.

### 2.3. Procedures and Experimental Conditions

Thresholds were determined for warble tones at 0.5, 1.0, 2.0, and 4.0 kHz, as well as for Speech Reception Threshold (SRT) using bisyllabic spondee words, following the modified Hughson–Westlake technique. SRTs were defined as the lowest intensity at which participants accurately repeated over 50 percent of the words. The BV was secured to the target sites using a 3 cm wide elastic rubber band stretched to its maximum capacity to maintain constant application pressure. Two distal sites were evaluated: the volar surface of the distal phalanx of the index finger and the medial arch sole of the right foot. The foot site was specifically chosen for its thicker soft tissue and its anatomical distance from underlying bone. Both the BV and the target body site were entirely submerged in the water bath during testing, ensuring no contact with the container walls. Both the stimulating BV and the target body sites (finger and foot) were submerged to a depth of approximately 5–7 cm below the water surface.

Four distinct experimental conditions were evaluated for each participant to validate the STC responses. The primary conditions involved direct contact between the submerged BV and either the finger or the foot. These were compared against two control conditions designed to assess the presence of non-contact artifacts. In the “in-water control”, the BV and the target limb were submerged with a gap of 8 to 10 cm between them to evaluate waterborne vibration (vibratory energy traveling through the water medium to the skin without direct contact). In the “out-of-water control,” the BV was submerged while the limb remained outside the bath to isolate residual AC leakage. During testing, participants were instructed to report tone perception or to repeat the words during SRT evaluation. If no response could be elicited at the maximum output of the audiometer (60dB HL for speech stimulus, 65 dB HL for 0.5 kHz; 85 dB HL for other frequencies), the threshold was recorded as 5 dB above the maximum intensity for statistical analysis.

### 2.4. Statistical Analysis

Statistical analysis was performed using R software (version 4.4.2; R Foundation for Statistical Computing, Vienna, Austria). Due to the small sample size (N = 10) and the expectation of high thresholds reaching the audiometer’s maximum output (ceiling effect)—particularly in control conditions based on previous distal hearing research—non-parametric statistical methods were employed. Data normality was assessed using the Shapiro–Wilk test. A Scheirer–Ray–Hare test was used to analyze test sites and stimulus frequencies. Significant main effects were followed by post hoc pairwise comparisons using Wilcoxon Signed-Rank tests. Across multiple comparisons, *p*-values were adjusted using the Bonferroni correction. A *p* < 0.05 was considered statistically significant.

## 3. Results

**Figure 1:**
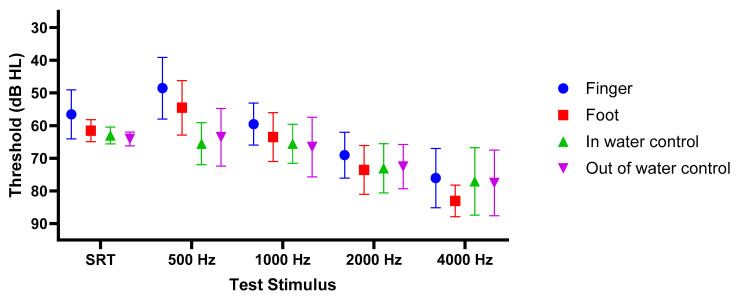
Mean tonal and speech recognition thresholds (SRT) (±SD) in dB HL. Thresholds are shown for the tip of the finger (blue circles), the foot (red squares), the “in-water” control (green upward triangles), and the “out-of-water” control (purple downward triangles). Stimulus intensities represent clinical audiometer dial settings in BC mode. In cases where no response was elicited at the maximal output of the audiometer, a value 5 dB greater than the maximum intensity was assigned for statistical purposes.

The primary analysis investigated the influence of stimulation site and frequency on auditory threshold as can be seen in Figure 1. As expected, the Shapiro–Wilk test indicated that the data was not normally distributed. The Scheirer–Ray–Hare test revealed significant main effects for both Test Site (H(3) = 21.49, *p* < 0.001) and stimulus frequency (H(4) = 168.97, *p* < 0.001). The interaction between site and frequency was not statistically significant (H(12) = 18.81, *p* = 0.093). Following this assessment, specific pairwise comparisons were performed using Wilcoxon Signed-Rank tests, with 95% confidence intervals (CI) reported for the observed differences.

### 3.1. Test Sites vs. Control Conditions

To verify that the recorded thresholds represent localized soft-tissue conduction rather than artifactual interference, thresholds at the target contact sites were compared with immersion controls. Post hoc pairwise comparisons revealed that at 0.5 kHz, thresholds at the finger site (48.5 dB, 95% CI: 41.7–55.3) were significantly lower than both controls (“in-water” control: 65.5 dB, 95% CI: 60.9–70.1; difference: −17.0 dB, *p* = 0.004; “out-of-water” control 63.5 dB, 95% CI: 57.2–69.8; difference: −15.0 dB, *p* = 0.006).

Finger SRT thresholds (56.5 dB, 95% CI: 51.2–61.8) showed trends toward lower values than controls (“in-water”: 63.0 dB, 95% CI: 61.2–64.8, *p* = 0.047; “out-of-water”: 64.0 dB, 95% CI: 62.5–65.5, *p* = 0.016); however, these differences were not statistically significant after Bonferroni correction.

At the foot site, 0.5 kHz thresholds (54.5 dB, 95% CI: 48.6–60.4) showed similar patterns (*p* = 0.014 and *p* = 0.031) without reaching corrected significance. For frequencies between 1 and 4 kHz, differences were reduced, with values approaching the maximum output limits of the B71 transducer.

### 3.2. Comparison of Finger and Foot Sites

A further analysis evaluated the influence of anatomical distance from the cochlea on STC sensitivity by comparing the two experimental sites. Thresholds at the fingertip (range across frequencies: 48.5–76.0 dB) were consistently lower than those at the foot (54.5–83.0 dB), though these differences did not reach statistical significance. The largest difference was observed at 4 kHz (finger: 76.0 dB, 95% CI: 69.5–82.5; foot: 83.0 dB, 95% CI: 79.5–86.5; mean difference: −7.0 dB, *p* = 0.125), followed by 0.5 kHz (finger: 48.5 dB, 95% CI: 41.7–55.3; foot: 54.5 dB, 95% CI: 48.6–60.4; mean difference: −6.0 dB, *p* = 0.086) and SRT (finger: 56.5 dB, 95% CI: 51.2–61.8; foot: 61.5 dB, 95% CI: 59.1–63.9; mean difference: −5.0 dB, *p* = 0.063).

### 3.3. Comparison of Control Conditions

Non-contact control conditions were evaluated to determine whether residual hearing was mediated by waterborne vibrations or AC leakage. No significant differences were found between the “in-water” and “out-of-water” control conditions across any of the measures (all *p* > 0.05). Average thresholds for both control conditions were near the maximum output of 65 dB at 0.5 kHz. At 1 kHz and 2 kHz, thresholds ranged from 55–80 dB. At 4 kHz, thresholds ranged from 60–85 dB with 55% of measurements reaching the equipment maximum.

## 4. Discussion

### 4.1. Key Findings and Physiological Interpretations

The findings of the present study indicate that the application of a clinical BV within a water bath was effective in reducing the effects of AC contamination at 0.5 kHz and SRT, enabling a more reliable isolation of distal STC thresholds. This allowed for the elicitation of STC responses at intensities below the AC thresholds measured within the same experimental setting. While this technique provides a preliminary methodological window into the physiological mechanisms of distal hearing [1], its efficacy appears to be frequency-dependent, showing optimal results in the lower spectral range.

The systematic threshold advantage of the fingertip over the foot aligns with physical laws of spatial attenuation and distance-dependent transmission loss [2]. While the small cohort (N = 10) likely limited the statistical power to reach significance at all frequencies, the consistent magnitude of these differences suggests a physiological trend. These findings may suggest that STC perception is determined by the distance the vibratory energy travels to reach the cochlea, providing preliminary support for the hypothesis that distal STC involves a localized propagation process through bodily tissues. In such a process, vibratory energy is progressively attenuated as it travels toward the inner ear.

Furthermore, participants’ ability to repeat bisyllabic words delivered via distal STC sites suggests potential activation of the entire auditory neural pathway. The successful verbalization of stimuli suggests that distal STC provides sufficient signal quality to support high-level cortical cognitive perception, clearly distinguishing these auditory responses from somatosensory sensations [12].

### 4.2. Comparison with Previous Literature

The efficacy of this method is further highlighted when compared to previous investigations of distal auditory thresholds. Brantberg et al. (2016) noted that in healthy subjects, thresholds obtained at distal sites (e.g., the ankle) were nearly identical to non-contact control thresholds at 0.5 kHz, concluding that they could not exclude AC hearing involvement [3]. While Verrecchia et al. (2023) subsequently utilized a specialized B250 transducer prototype to evaluate the clinical feasibility of ankle audiometry, their study did not explicitly describe non-contact control conditions to definitively differentiate STC from potential airborne sound involvement [6]. In contrast, our findings at 0.5 kHz demonstrate a significant separation between foot and finger thresholds compared to control conditions. This robust distinction suggests that the water-immersion setup successfully provides the acoustic shielding necessary to isolate pure STC hearing, overcoming the AC contamination issues that limited earlier studies using standard clinical equipment.

### 4.3. Clinical Implications

The clinical implications of these findings are potentially significant for the systematic evaluation of “third window” pathologies. Clinical reports have long suggested that applying a tuning fork to distal sites, such as the ankle, can elicit a hearing sensation in patients with suspected SCDS [13,19]. However, these reports have largely remained anecdotal and have not been systematically validated due to methodological challenges in isolating STC from AC at the required intensities. Previous systematic attempts to address these challenges, such as the work by Brantberg et al. [3] and Verrecchia et al. [6], required specialized equipment, such as mini-shakers or expensive prototypes (e.g., the B250 transducer), which are not typically available in standard clinical facilities. Furthermore, earlier studies often lacked the rigorous non-contact control conditions required to definitively exclude residual air-conduction.

The results of this preliminary study suggest that distal STC can be reliably elicited and isolated using standard clinical bone vibrators, particularly at 0.5 kHz and with SRT measures. If further validated, a water-immersion setup could provide clinicians with a practical method for differentiating true STC responses from air-conduction artifacts without specialized hardware. Such an approach might eventually contribute to the development of accessible screening protocols for SCDS, potentially offering a pathway for broader clinical participation in the specialized diagnosis of vestibular–auditory pathologies within the existing clinical setting.

### 4.4. Strengths

A potential strength of this study lies in its methodological rigor, notably the employment of two distinct non-contact control conditions: “in-water” and “out-of-water”. Results showed that thresholds during both immersion controls were significantly higher than those measured during direct skin contact, particularly at 0.5 kHz and for SRT measurements. This finding provides evidence suggesting that auditory perception in the immersion condition was likely mediated by point-contact STC, rather than residual AC waterborne vibration. Furthermore, the lack of a significant difference between the two control conditions indicates that residual perception was primarily due to AC sound leaking from the water surface. Had vibrations been effectively transmitted through the water to stimulate the submerged limb, lower thresholds would have been expected in the “in-water” control; their parity instead suggests that AC leakage determines the absolute threshold floor of the system. Additionally, the successful isolation of SRT thresholds provides functional evidence that the STC pathway can transmit cognitively processable auditory information. Finally, the reliance on common clinical equipment (Radioear B71) ensures that the method is easily replicable in standard facilities without requiring specialized laboratory hardware.

### 4.5. Limitation

Several limitations warrant consideration. First, the small sample size (N = 10) limited the statistical power to detect significance across all frequencies. Second, the reliance on a single-center cohort of young, healthy participants restricts the immediate generalizability of the findings to older individuals, where tissue density and body composition may vary. Consequently, these results should be viewed as preliminary and warrant further validation in larger, more diverse cohorts.

Technical constraints also impacted the data. While water immersion provides significant acoustic shielding, it does not achieve complete spectral isolation. At higher frequencies, the gap between the contact and control thresholds narrowed, likely due to the B71 transducer’s output limits or mechanical resonances in the water bath. These factors complicate the measurement of high-frequency distal conduction, as standard clinical equipment lacks the intensity required to clearly separate STC from residual AC noise in this range. Finally, measurements were restricted to only two anatomical sites (finger and foot), leaving a comprehensive mapping of auditory conductivity across the entire body for future investigation.

## 5. Conclusions

In conclusion, while the present study was limited to the fingertip and the foot, the findings provide preliminary evidence that water immersion is a potentially valid and accessible technique for the study of distal auditory physiology. Future research involving a wider array of body sites and equipment with a broader dynamic range, and larger cohort also including older subjects, will be essential to further map the body’s auditory conductivity and to confirm whether thresholds at various distances consistently reflect the inverse-square law.

## Data Availability

The data presented in this study are available on request from the corresponding author due to privacy and ethical restrictions.

## Abbreviations

The following abbreviations are used in this manuscript:

STC	Soft Tissue Conduction
BV	Bone Vibrator
AC	Air Conduction
BC	Bone Conduction
SRT	Speech Reception Threshold
